# Presence of pseudo-Kayser-Fleischer rings in patients without Wilson disease: a prospective cohort study

**DOI:** 10.1097/HC9.0000000000000136

**Published:** 2023-04-14

**Authors:** Swapnali Sabhapandit, Anand Kulkarni, TR Soumya, Shireesha Anumula, Mounika S. Konda, Tumula A. Shaik, Perumalla Solomon, Padaki N. Rao, Nitin Jagtap, Duvvur N. Reddy, Mithun Sharma

**Affiliations:** 1 Institute of Ophthalmic Sciences, AIG Hospitals, Hyderabad, India; 2 Department of Hepatology and Liver Transplantation, AIG Hospitals, Hyderabad, India; 3 Department of Gastroenterology, AIG Hospitals, Hyderabad, India; 4 Department of Hepatology, AIG Hospitals, Hyderabad, India

## Abstract

Kayser-Fleischer (KF) rings are due to copper deposition in Descemet membrane of the cornea in Wilson disease. Pseudo-KF ring (PKF), seen in patients with high bilirubin, is often misinterpreted as KF rings. These are bilirubin deposits in posterior corneal stroma. The aim of the study was to prospectively evaluate the presence of KF and PKF rings in Wilson disease and non-Wilsonian liver disease with serum bilirubin >5 mg/dL.

**Methods:**

All patients referred from the hepatology unit with serum bilirubin >5 mg/dL were assessed by slit-lamp examination (SLE) and anterior segment optical coherence tomography at baseline, 3 months, and 6 months for differences in corneal deposits between KF and PKF rings. All other clinical, laboratory, radiological, genetic, and tissue diagnoses by liver biopsy were done as required to confirm the cause of liver disease.

**Results:**

Among the 750 patients examined, corneal deposits were present in 13%, KF rings as granular deposits in 31.7%, and PKF as a posterior stromal hue in 68.3% of cases. PKF rings showed regression in 60%, disappearance in 36.6% at 3 months, and in 100% of cases at 6 months. KF ring showed regression in 10.7% and 8.3% until 6 months. Anterior segment optical coherence tomography identified KF ring as a hyperintense line on Descemet membrane in an additional 9.7% of patients compared with a scattered hyperintense hue in PKF rings.

**Conclusions:**

The presence of PKF rings in patients with jaundice is not uncommon and should be differentiated from true KF rings. Serial monitoring is essential to look for resolution, and anterior segment optical coherence tomography may be additionally helpful.

## Graphical abstract

**Figure:**
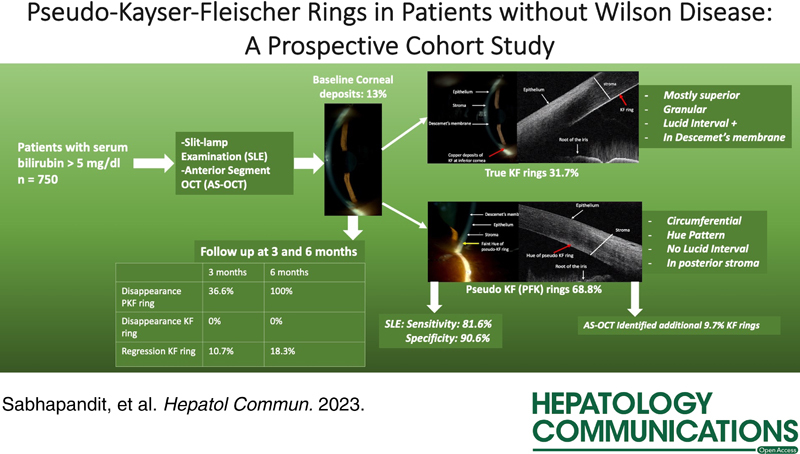

## INTRODUCTION

Wilson disease (WD) is a rare liver disease resulting from defective biliary excretion of copper, leading to abnormal copper depositions in different body organs.^1^ Abnormal copper deposition can occur in the liver, brain, and other body tissues, including the eyes. It occurs owing to mutations in the ATP7B gene on chromosome 13.^2^ The prevalence of this disease is 1:30,000–1:50,000 per live birth.^3^

The clinical presentation of WD is extremely varied, ranging from asymptomatic detection to presentation with jaundice, acute liver failure (ALF), and neurological features, which can be subtle or overt manifestations like tremors and dyskinesia.^4^ Some patients can present with only psychiatric manifestations like depression, insomnia, and even psychosis.^5^

The Leipzig score is commonly used to diagnose WD in adults and children. This score includes specific clinical features like the presence of KF rings, Coombs-negative hemolytic anemia, neuropsychiatric manifestation, and imaging brain features of WD with laboratory findings of 24-hour urinary copper excretion, serum ceruloplasmin levels, and estimation and staining for copper on liver tissue after liver biopsy. Genetic analysis for ATP7B gene mutation is also included in this score. A score of >4 suggests a high likelihood of WD, whereas scores of ≤1 make the diagnosis a remote possibility.^6^ However, in a real-life scenario, all these tests, including liver biopsy and genetic test, may not be required to diagnose WD.

The Kayser-Fleischer (KF) ring is considered a pathognomonic finding in WD and is present in 95% of patients with neurological and 50% of hepatic WD^7^ on slit-lamp examination (SLE). It is described as a pigmented golden brown or greenish-brown ring because of copper deposition in Descemet membrane (DM) of the peripheral cornea.^8^ The finding is primarily bilateral, with a predilection for the superior and inferior areas, which may become circumferential as the disease progresses. KF rings can be diagnosed by SLE, gonioscopy, anterior segment optical coherence tomography (AS-OCT), and in vivo confocal microscopy.^9^,^10^

KF rings usually indicate a longer duration of the disease but may be absent in pediatric patients and 50% of patients with fulminant hepatic failure.^11^ Presence of KF rings should prompt the physician to search for additional clinical and imaging signs of WD. Patients with absent KF rings who present with low ceruloplasmin and abnormal liver tests should be considered for liver biopsy.^12^

Corneal deposits are found in non-Wilsonian conditions such as primary biliary cholangitis (PBC), autoimmune hepatitis, hepatic malignancy, and cryptogenic cirrhosis, where the deposits are bilirubin.^13^–^15^ The differentiation between pseudo-KF (PKF) and true KF rings is difficult for even an experienced ophthalmologist. A liver biopsy with dry copper weight estimation and genetic analysis for the ATP7B gene mutation help with diagnosis.^16^

The current study evaluates the prevalence of KF and PKF rings in patients with icterus who had a serum bilirubin of >5 mg/dL. Differences between KF and PKF rings were studied.

## METHODS

This was a prospective, observational study of consecutive patients with jaundice with serum bilirubin of >5 mg/dL at presentation or patients with clinically suspected WD referred for KF rings evaluation from the hepatology unit of the institute. The study period was between January 2020 and December 2021.

The research was conducted in accordance with both the Declarations of Helsinki and Istanbul. The Asian Institute of Gastroenterology Institutional Review Board approved the study. Informed written consent was obtained from all study participants.

The inclusion criteria of the study were as follows:

1. Patients of any age with serum bilirubin >5 mg/dL where the anatomical cause of obstructive jaundice in the intrahepatic or extrahepatic biliary system was ruled out.
2. Patients of any age who were clinically suspected of having WD as defined by serum ceruloplasmin <20 mmol/L and/or 24 hours urinary copper >40 mcg who were referred for KF ring evaluation.^17^
3. Patients with ALF of any etiology having serum bilirubin >5 mg/dL.
4. Patients with jaundice with Coombs-negative hemolytic anemia.

Patients with a history of copper foreign body in intraocular structures or cornea, occupational copper exposure, topical use of copper-containing substances, pregnant women, and impaired ocular fixation were excluded from the study. Patients whose cause of jaundice was found during initial evaluation to be due to anatomical obstruction of the biliary tree were also excluded from the study.

In the Institute of Ophthalmic Sciences, all patients underwent a detailed evaluation, including documentation of the history of the disease with duration, visual acuity testing, applanation tonometry, SLE of the anterior segment, and detailed fundus and optic nerve head examination after instillation of mydriatic eye drops for both eyes (tropicamide 0.8% with phenylephrine 5% for both eyes). The SLE was conducted by 2 trained ophthalmologists (one of them—Swapnali Sabhapandit, a trained cornea and anterior segment specialist) independently, and findings were noted individually by each of them. Gonioscopy was done in patients with high clinical suspicion and absent KF rings. A hand-held slit lamp was used in 2 patients who were too sick to undergo a routine SLE at baseline presentation. AS-OCT was done in all patients.

Baseline complete blood counts, liver function test, kidney function test, serum copper, ceruloplasmin, 24-hour urinary copper, antinuclear antibody, anti–smooth muscle antibody, antimicrobial antibody, and tests for hepatitis A, B, E, and C were done for all patients. Imaging tests included ultrasonography of the abdomen with portal venous Doppler, magnetic resonance cholangiopancreatography, endoscopic ultrasonography, and contrast-enhanced computerized topography as and when indicated. In patients with neurological involvement like tremors and tardive dyskinesia, MRI of the brain was done to look for T2 hyperintensity in the thalamus, putamen, and brainstem.^18^

The presence of golden brown or greenish-brown deposits at the sclero-corneal junction on DM of the cornea on SLE identified KF ring. Diagnosis of the PKF ring was made by the presence of a yellow to yellowish-green hue rather than deposits at the posterior stromal level of the cornea by SLE. This was later clinically confirmed as a PKF ring if the patient had no other evidence of WD.

A presumptive diagnosis of WD was made if 2 of the following 3 criteria were present:

1. Serum ceruloplasmin <20.
2. 24-hour urinary copper >2 times the upper limit of normal.
3. Presence of KF ring on SLE.

A liver biopsy with dry copper weight estimation was done in 5 patients, and a cutoff of >250 μg/g dry copper weight was considered positive. Genetic analysis of ATP7B mutation was done in 10 patients.^12^ The Leipzig scoring system was used to confirm further the diagnosis of WD in patients in whom genetic analysis was done. A score of >4 was considered diagnostic of WD, whereas those between 2 and 3 had probable WD.^19^

Ophthalmic examination was repeated at 3 and 6 months on follow-up. All other laboratory parameters were done at regular intervals as required for managing the underlying liver disease. The first-degree relatives who were available with the patients were screened for corneal deposits by SLE.

### Statistical analysis

The data were collected using a study proforma and entered into Microsoft Excel for analysis. The continuous variables were expressed as mean and SD for parametric data and median and interquartile range for nonparametric data. The categorical data were expressed as a percentage.

## RESULTS

**FIGURE 1:**
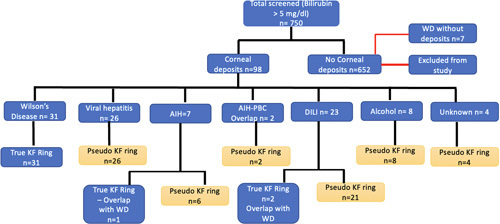
Flowchart showing patients of different etiology with corneal deposits (black lines) and patients with Wilson disease without KF rings (red line). Abbreviations: AIH, autoimmune hepatitis; KF, Kayser-Fleischer; PBC, primary biliary cholangitis; WD, Wilson disease; n, number of patients.

A total of 750 patients with serum bilirubin >5 mg/dL were screened for corneal deposits by SLE. Corneal deposits (KF ring and PKF ring) were found in 98/750 (13%) of patients. Of the remaining 652 patients who did not have corneal deposits, 7 (1.1%) were still diagnosed with WD, and 2 with KF-negative WD were in ALF (Figure 1).

Of the 98 patients with corneal deposits, 31 (31.6%) had true KF rings, and 67 (68.3%) had PKF rings. Of the 750 screened patients in this tertiary care center, 31 (4%) had true KF ring considered pathognomic of WD. The baseline characteristics of the patients with KF and PKF rings are shown in Table 1.

**TABLE 1:** The cause of jaundice in patients with KF and pseudo-KF rings

	KF ring n=31	Pseudo-KF ring n=67
Male:female (n)	26:5	48:19
Age of presentation (mean±SD)	24.6±12.4	28.4±7.6
Median duration of jaundice (median, range) in days	24 (10–86)	18 (7–110)
Peak serum bilirubin (mg/dL)	29.7	30.4
Ceruloplasmin mg/dL (median/range)	16 (8–20)	24 (19–28)
24 h urinary copper μg/dL (median, range)	108 (64–268)	48 (26–78)
Underlying cirrhosis of liver, n (%)	3 (9.67)	11 (16.4)
Cause of jaundice		
Wilson disease	31	0
Viral hepatitis	0	26
AIH	1 (simplified AIH score 6)—probable AIH	6
AIH-PBC overlap	0	2
DILI	2	21
Alcohol-associated hepatitis	0	8
Unknown	0	4

Abbreviations: AIH, autoimmune hepatitis; AIH-PBC, autoimmune hepatitis-primary biliary cholangitis overlap.

Among the 31 patients diagnosed with WD, 20 (64.5%) had components of both hepatic and neurological WD. Tremor and dyskinesia were found in 11 patients with neurological WD. T2 hyperintensity on MRI of the brain in the region of the hippocampus and lentiform nucleus was found in 15 (75%) patients. Four patients presented with ALF, and 2 of them died during the first admission. One patient with WD-ALF underwent living donor liver transplantation, and another recovered with conservative treatment and chelation with triethylenetetramine dihydrochloride.

All 20 (100%) patients with both hepatic and neurological WD had positive KF rings on SLE, whereas 7/11 (63.6%) of pure hepatic WD had KF rings on SLE. In three fourth of patients of pure hepatic WD where SLE did not detect KF rings, AS-OCT demonstrated KF rings.

In the cohort of patients with KF rings and WD, 1 patient had an overlap of features of probable autoimmune hepatitis (simplified autoimmune hepatitis score of 6), whereas 2 patients had superimposed DILI features (bile cholestasis, 15%–20% microvesicular fat, and increased eosinophils) on liver biopsy due to intake of complementary and alternative medicine.

**FIGURE 2:**
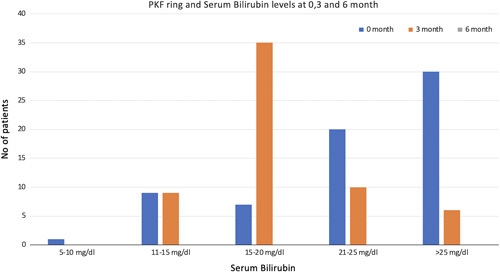
Figure showing the disappearance of PKF rings based on the time duration and level of serum bilirubin. Abbreviation: PKF, pseudo-KF ring.

The progression, stable pattern, or regression of the corneal deposits decreased with the decreasing trend of serum bilirubin level in patients with the PKF ring but not with WD (Figure 2A). Regression was considered if there was at least a 25% decrease in the intensity of the rings and/or regression from either of the quadrants of the cornea when compared with the baseline SLE. Table 2 shows the status of the corneal deposits at baseline, 3 months, and 6 months.

**TABLE 2:** Baseline presence of corneal deposits and its progression at 3 months and 6 months

	Baseline		3 mo		6 mo	
	True KF ring (n=31)	Pseudo-KF ring (n=67)	True KF ring (n=28)	Pseudo-KF ring (n=60)	True KF ring (n=24)	Pseudo-KF ring (n=55)
Baseline serum bilirubin (mg/dL)	15.34±14.2	20.38±9.92	8.8±6.4	13.2±7.2	3.0±2.4	3.2±1.3
Regression of corneal deposits (%)	—	—	10.7	68.3	19	100
Disappearance of corneal deposits (%)	—	—	0	68.3	0	100
Same intensity of corneal deposits	—	—	89.3	5	81	0

*Note:* True KF ring: golden brown or greenish-brown granular corneal deposits of copper in patients with Wilson disease; Pseudo-KF ring: yellow to the yellowish-green nongranular hue of bilirubin in the cornea in non-Wilsonian liver disease on slit-lamp examination.

Abbreviations: KF, Kayser-Fleischer ring; n, number.

During the follow-up of these patients, at the end of 3 months, 2 patients with WD succumbed to the disease (both presented with ALF), and 1 patient was lost to follow-up. Three patients died in the non-wilson disease (NWD) group, and 4 were lost to follow-up. Of 28 patients of WD who were initiated with chelation therapy, 3 (10.7%) showed regression of KF rings. In comparison, 36/60 (60%) patients with PKF rings showed regression, 22/60(36.6%) showed disappearance, and 2/40 (5%) had no change in the intensity of corneal deposits on SLE (Figure 2A).

In the follow-up period between 3 and 6 months, 2 patients with WD were successfully transplanted, and the other 2 cases were lost to follow-up and were excluded from the analysis. In the NWD group, at the end of 6 months, 2 patients died because of progressive liver disease and sepsis, whereas 3 more were lost to follow-up. SLE at the end of 6 months showed the disappearance of the corneal deposits in all patients with NWD, whereas further regression of the KF ring was observed in 2/24 (8.3%) patients on chelation therapy. No patient in the WD group had a disappearance of KF rings (Figure 2B).

SLE and AS-OCT images of KF and PKF rings were studied to observe the difference in the deposits’ location, color, pattern, depth, and intensity (Table 3).

**TABLE 3:** The clinical and imaging differences between true and pseudo-KF rings

Features	KF ring	Pseudo-KF ring
Location	Superior>inferior>circumferential	Circumferential
Depth of penetration in the cornea	Descemet membrane	Posterior stroma (poorly captured by slit-lamp imaging but better visualized on examination)
Lucid interval	Yes	No
Consistency	Granular	Smooth/hue
Correlation with serum bilirubin	Occasionally	Always
Correlation with chelation therapy	Yes	Not applicable
Presence of deposits in the cornea of family members	Possible (3.2%)	No
Color	Golden brown/greenish brown	Yellow/yellowish green
Visible to the naked eye	Yes	No
AS-OCT finding	Hyperintensity at Descemet membrane	Scattered hyperintense hue at stroma

Abbreviations: AS-OCT, anterior segment optical coherence tomography; KF, Kayser-Fleischer.

**FIGURE 3:**
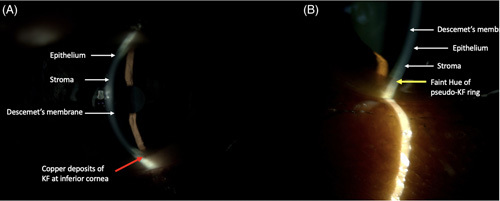
Figure showing deposits in the cornea. Red arrow in (A) shows copper deposits of the KF ring, whereas in (B), the yellow arrow shows the hue of bilirubin deposit in the posterior stroma, which is better appreciated on slit-lamp evaluation then captured on imaging. Abbreviation: KF, Kayser-Fleischer.

Granular golden brown or greenish-brown deposits of KF rings were detected at the superior corneal limbus (80%), followed by the inferior (16%) and circumferential (4%) parts of the cornea (Figure 3A, B). On the contrary, smooth, nongranular deposits of PKF rings had a yellow to yellowish-green hue, which was better visualized than recorded on SLE. These were predominantly circumferential (90%), with involvement of the superior quadrant and inferior quadrant in 7% and 3% of cases, respectively (Figure 3A).

The Lucid interval, defined as a clear space between the limbus and the corneal deposit, was noted in KF rings and not in PKF rings with circumferential deposits.

**FIGURE 4:**
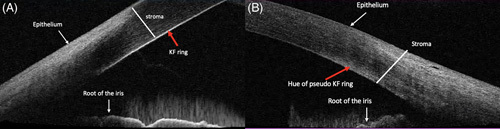
Figure showing hyperintense deposit of copper on Descemet membrane of the cornea (red arrow) in (A), whereas in pseudo-KF ring, there is a hyper-reflective hue (B, yellow arrow). Abbreviation: KF, Kayser-Fleischer.

On AS-OCT, KF rings appeared as a hyperintense line in the DM, whereas PKF rings appeared as a scattered hyperintense hue on the posterior stroma (Figure 4).

On gonioscopy, the true KF ring was seen as a sharp line pattern at Schwalbe line limited to DM, whereas the deposits of the PKF ring involved the central cornea to a depth of around 5 mm in the posterior stroma.

The sensitivity of KF ring on SLE for WD diagnosis was 81.6% (95% CI, 65.7%–92.2%), whereas the specificity was 90.6% (95% CI, 88.2%–92.6%). The positive and negative predictive values KF ring for the presence of WD were 31.6% (95% CI, 26.0%–37.8%) and 98.9% (95% CI, 97.9%–99.4%), respectively. The CI for predictive values is the standard logit CI.^20^

Screening of the first-degree relatives did not reveal any findings in the PKF ring arm, whereas 1/31 (3.2%) (sibling of a patient with ALF) had an asymptomatic KF ring and was diagnosed with WD. Chelation therapy was initiated for this subject.

## DISCUSSION

KF rings on SLE have been long considered the eureka moment in the diagnosis of WD. However, corneal deposits can be present in many non-Wilsonian liver diseases. True KF rings are present in only 60% of cases with hepatic WD and much higher (90.4%–100%) in patients with neurological WD.^7^,^21^,^22^

### Sensitivity of KF ring for diagnosis of WD

In our current study, all patients with neurological WD had KF rings. In comparison, it was present in 63.7% of hepatic WD. In addition, 9.7% of hepatic WD had KF rings diagnosed by AS-OCT but were not visible on SLE. The sensitivity of SLE, when compared with AS-OCT, is low for detecting early KF rings and also depends on the skill of the ophthalmologist.^23^,^24^ Therefore, all cases in this study were evaluated by 2 independent ophthalmologists, 1 of whom was a trained cornea specialist. Despite this, in 3 patients, SLE missed the KF rings. AS-OCT and gonioscopy can identify these missed cases in up to 62% of patients with early WD and normal SLE.^9^,^23^ The sensitivity of SLE for WD in the current study was diagnosis was 81.6%, whereas the specificity was 90.6%.

### PKF rings in non-Wilsonian disease

PKF rings or KF-like rings can be present in many conditions, which can be confused with true KF rings and lead to an erroneous diagnosis of WD. These conditions include cholestatic liver diseases like PBC, hepatitis A, autoimmune hepatitis, and cryptogenic cirrhosis.^15^,^25^–^27^ In all patients with PKF rings, there is either deposition of bile pigments or even copper. In PBC, where the excretion of copper through the biliary tree is blocked, the levels of copper in the liver increase.^28^ Patients with PBC may demonstrate very high levels of copper in the liver and blood with increased urinary excretion of copper. In this scenario, a normal to high serum ceruloplasmin and the ability to differentiate true KF rings from PKF rings become essential.

### Cutoff of serum bilirubin for PKF rings

The current study aimed to find the difference in interpretation of the ophthalmologic findings correlating with the background hepatic parameters and suggested the difference between the 2 types of deposits. On the basis of previous studies, which found that PKF rings disappear in most patients at serum bilirubin <10 mg/dL, we took a cutoff value of 5 mg/dL for serum bilirubin.^13^,^29^ The lower cutoff of 5 mg/dL serum bilirubin was taken to account for any cases that may have been missed. However, none of the patients had PKF rings with serum bilirubin <5 mg/dL.

### Disappearance or regression of PKF rings

PKF or KF-like rings have been previously reported in patients with jaundice and serum bilirubin >10 mg/dL in up to 50.7% of patients, whereas in our study, it was found in a much higher proportion (68.3%).^28^ PKF rings are found only in patients with serum bilirubin of 10 mg/dL or higher in previous studies. These PKF rings were present in only 60% of cases with reduced intensity, whereas there was a disappearance in 33.6% and 100% of patients at 3 and 6 months, respectively. These data differ from the previous study, where the PKF rings disappeared in 86.6% of cases.^13^ This may be attributed to the duration of follow-up of these patients in different studies. The disappearance of the PKF ring occurs in all patients with a decrease in jaundice, usually <10 mg/dL^29^. A repeat examination of all patients documented to have a KF ring is required once the bilirubin level decreases.

### Disappearance or regression of true KF rings

The regression and disappearance of the intensity of true KF rings have been reported and corelate with the response to chelation therapy or post–liver transplantation in patients with WD.^1^,^30^,^31^ The regression of KF rings have been reported between 41% and 90% of cases of WD^32^,^33^. However, the disappearance of a true KF ring takes a longer time when compared with a PKF ring, which disappears in 100% of patients with the resolution of jaundice.^33^

### Regression pattern of true versus PKF rings

The regression of KF rings has been reported to start initially from the lateral segments. The possible explanation is increased photochemical degradation that makes the copper more amendable to chelation by penicillamine as the upper and lower segments are usually shielded from sunlight by the eyelids.^34^ No such regression pattern has been observed in patients with PKF rings. In our study, regression of PKF ring paralleled the levels of serum bilirubin, and all patients with bilirubin <5 g/dL on treatment had a disappearance of PKF rings.

### KF versus PKF: different pathogenesis and location

The difference between corneal deposits in KF rings and PKF rings is likely related to the primary underlying pathogenic mechanism. True KF rings start superiorly in the cornea and then progressively involve the inferior and horizontal zone of the cornea. This is attributed to the flow of aqueous humor in the eye’s anterior chamber.^35^ Patients with true KF rings have significantly more central and posterior corneal densitometries.^36^ On the contrary, PKF rings are mostly circumferential with no lucid interval between the limbus and the cornea. These deposits are due to the endothelial seepage of bilirubin from the limbal circulation.

In patients with PBC who have corneal deposits, it is still not sure whether the deposits are due to increased copper or bilirubin, as cases of PBC with normal bilirubin have been reported to have PKF rings rarely.^25^

### Pattern of deposits in KF and PKF ring

The granular pattern of deposits of copper has been reported extensively in patients with KF rings in WD. The granular deposits are arranged in layers with the smallest granules near the endothelium, suggesting a role of endothelial absorption of copper from the aqueous humor.^32^ Higher levels of copper have been found in the aqueous humor in such cases.^37^ Ultrastructure analysis of corneal biopsies in patients of WD having KF ring has failed to demonstrate a consistent deposition pattern as the number of cases for such analysis has been minimal.^38^ Whether the PKF rings are depositions in the stroma or DM has contrasting views.^13^,^29^

### Limitations of the study

The study had some limitations. First, we did not consider the fractionalization of serum bilirubin in these patients at the time of enrollment. Indirect hyperbilirubinemia, which can be attributed to hemolysis in WD, was also difficult to ascertain as many of these patients were from a geographic region where the prevalence of Gilbert syndrome and beta-thalassemia trait was high. Second, genetic analysis was not performed in all the cases. In addition, the study was not powered to see the correlation of disappearance or regression of the PKF ring with a linear observation of serum bilirubin levels. Finally, pregnant patients were not included in the study because of logistic issues of managing these patients in a center without regular obstetric services.

## CONCLUSIONS

Corneal deposits are common in patients with cholestatic jaundice of any etiology, and it is essential to differentiate the true KF ring from the PKF ring, which can be challenging. KF rings are present in many NWDs. Hence, a clinical and biochemical correlation of the ophthalmic findings is extremely important. Increased use of AS-OCT may help identify missed cases of KF rings.
